# Combination of hypoxia and hyperosmolarity reduces *in vitro* chondrocyte de-differentiation

**DOI:** 10.3389/fvets.2026.1802356

**Published:** 2026-03-25

**Authors:** Elena De Angelis, Roberta Saleri, Filippo Marcotti, Luca Ferrari, Valeria Cavalli, Rosanna Di Lecce, Matteo Zoboli, Francesca Ravanetti, Benedetta Passeri, Paolo Martelli, Paolo Borghetti

**Affiliations:** Department of Veterinary Science, University of Parma, Parma, Italy

**Keywords:** articular cartilage, chondrocytes, differentiation, hypoxia, osmolarity

## Abstract

**Introduction:**

Articular cartilage (AC) is an avascular tissue with a highly specialized extracellular matrix (ECM) microenvironment characterized by low oxygen tension and high osmolarity. Standard in vitro culture conditions fail to replicate these features and may promote chondrocyte de-differentiation. This study investigated the combined effects of hypoxia and hyperosmolarity on chondrocyte phenotype and function.

**Methods:**

Human articular chondrocytes were cultured under standard conditions (20% O_2_, 280 mOsm/L) or under cartilage-mimicking conditions (5% O_2_, 380–480 mOsm/L). Cells were maintained in two-dimensional (2D) monolayers and three-dimensional (3D) alginate bead cultures. Gene expression of differentiation markers (*COLL2A1*, *ACAN*, *SOX9*), de-differentiation markers (*COLL1A1*, *RUNX2*), and adaptive markers (*HIF-1a*, *BGT1*) was assessed. Glycosaminoglycan (GAG) production was quantified across passages.

**Results:**

Hypoxia and hyperosmolarity synergistically reduced de-differentiation marker expression and enhanced *COLL2A1* expression, particularly during early passages. This effect was more pronounced in 3D cultures. Hyperosmolarity increased GAG production across passages, while its combination with hypoxia showed a synergistic effect in early 2D cultures and consistently in 3D systems. *HIF-1a* expression was upregulated under combined conditions. However, these protective effects decreased at later passages as de-differentiation progressed.

**Discussion:**

Mimicking the native cartilage microenvironment modulates chondrocyte phenotype and function, particularly in early de-differentiation stages. Although responsiveness declines over time, key phenotypic markers remain influenced. These findings support the importance of physiologically relevant culture conditions to improve chondrocyte quality for cartilage repair strategies, including autologous chondrocyte implantation (ACI) and matrix-assisted chondrocyte implantation (MACI).

## 1 Introduction

The prevalence and clinical relevance of osteoarthritis (OA) have markedly increased as a consequence of population aging and the progressive extension of life expectancy in both humans and animals. Despite extensive research efforts, OA treatment remains a major clinical challenge, largely due to its complex and still not fully understood pathogenesis, which involves profound alterations in chondrocyte phenotype, including progressive de-differentiation during disease progression (55).

This limited understanding hampers the development of effective therapeutic strategies and delays the clinical translation of tissue engineering approaches aimed at restoring native cartilage structure and function.

Cell-based therapies for cartilage repair, such as autologous chondrocyte implantation (ACI) and matrix-assisted chondrocyte implantation (MACI), require extensive *in vitro* expansion to obtain a sufficient number of cells for implantation. However, during serial monolayer culture, adult articular chondrocytes progressively lose their differentiated phenotype—a process known as *in vitro* de-differentiation—which represents a major limitation for the success of these therapies (1).

Consequently, current research efforts are focused on defining the optimal microenvironmental conditions that allow chondrocytes to maintain their differentiated phenotype, minimize de-differentiation during *in vitro* culture, or alternatively optimize inefficient re-differentiation protocols (1–6).

The mechanical role of articular cartilage in synovial joints is to reduce and dissipate compressive and tensile mechanical forces generated during locomotion (7) and is highly dependent on the molecular organization of the cartilage extracellular matrix (ECM). Large aggregating proteoglycans (LAPs), located inside the collagen type II network, retain high volumes of water and cations (Na^+^, Ca^2+^, and H^+^), creating an internal hydrostatic pressure that counteracts external mechanical loads. As a result of the high concentration of retained cations, the osmolarity of cartilage ECM is higher than the synovial fluid and other physiological fluids: articular chondrocytes are typically adapted to 350–450 mOsm/L (8–10). The cartilage ECM can also respond to further increases of extracellular osmolarity occurring when compressive loading is applied (9, 11).

The cartilage tissue is avascular, therefore O_2_ concentration is lower than in other tissues; for this reason, *in vivo* chondrocytes are adapted to sustained hypoxic conditions (typically ranging from 1 to 10% O_2_ tension). Hypoxia represents another crucial micro-environmental factor with a known positive effect on chondrocyte maintenance and differentiation (12–15).

Therefore, a micro-environment characterized by high osmolarity and low O_2_ tension appears to be a pre-requisite for chondrocyte differentiation, metabolic activity and ECM production (7, 16–20).

In addition, mature chondrocytes within articular cartilage typically exhibit a round shape; this morphology is tightly linked to their differentiated phenotype characterized by the expression of cartilage-specific markers such as type II collagen (*COLL2A1*), aggrecan (*ACAN*), and SRY-box transcription factor 9 (*SOX9*), whereas type I collagen and runt-related transcription factor 2 (*COLL1A1*, *RUNX2*) are induced upon the de-differentiation process (1, 21, 22). This phenotype supports their primary role in maintaining tissue homoeostasis, specific ECM composition and mechanical integrity of cartilage (23).

In the present work, we investigated the response of horse articular chondrocytes to osmolarity and O_2_ tension changes both during de-differentiation in monolayer (adhesion) culture (1, 3) and when chondrocytes are cultured in a 3D alginate system that represents an *in vitro* model to maintain the chondrocyte phenotype. The differentiation potential was determined in terms of gene expression of differentiation markers and GAG production. Chondrocyte differentiation status was evaluated through gene expression of differentiation (*COLL2A1, ACAN, SOX9*) and de-differentiation markers (*COLL1A1* and *RUNX2*), as well as markers associated with the adaptive response to hypoxia (*HIF-1α*) and osmolarity (*BGT1*).

## 2 Materials and methods

### 2.1 Media formulation

The osmolarity of the standard medium (complete medium: D-MEM supplemented with 4.5 g/L glucose, 25 mM Hepes, 50 μg/mL ascorbic acid, 100 U/mL penicillin, 0.1 mg/mL streptomycin, 0.25 μg/mL amphotericin B, and 10% fetal bovine serum [FBS] [Merck, Darmstadt, Germany]) was adjusted from an iso-osmotic value of 280 (286 ± 4.2) mOsm/L to hyperosmotic values of 380 (384 ± 4) mOsm/L and 480 (482 ± 2.9) mOsm/L by the addition of a sodium chloride (NaCl) solution (Merck), and measured with a Wescor 5,500 vapor-pressure osmometer (VPO) (Wescor, Utah, USA).

### 2.2 Cartilage explant

Chondrocytes were isolated from articular cartilage explanted from 12 fetlock joints collected from adult horses (5–8 years old) without any distinction on sex or breed, regularly slaughtered for human consumption in a slaughterhouse certified by the Italian Ministry of Health according to the Regulation (EC) 853/2004 (Zerbini&Ragazzi S. R. L., Correggio (Reggio Emilia), Italy; approval nr. CE-IT 798-M) and delivered to the laboratory within 1 h 30 min. under controlled conditions (1, 24).

The joints were opened under sterile conditions in a laminar flow hood to expose the two joint heads. The conditions of the joints were first evaluated, discarding the joints that presented blood in the synovial fluid, osteoarthritic lesions or other pathological conditions, as they were not suitable for chondrocyte isolation.

The articular surfaces were continuously hydrated with sterile phosphate buffer saline (PBS, Merck) containing amphotericin B and penicillin/streptomycin (2x) in order to reduce any contamination and tissue dehydration. A scalpel was used to incise a grid on the cartilage, which allowed small squares of cartilage tissue to be collected.

The cartilage pieces were pooled (4 joints/experiment) and then placed into cell culture plates containing sterile PBS with amphotericin B and penicillin/streptomycin (2x), and subsequently washed 3 times with the same solution.

### 2.3 Chondrocyte isolation

Cartilage pieces were then transferred into a sterile 50 mL tube with a 0.1% protease solution (Merck) in serum-free D-MEM and incubated at 37 °C for 1 h.

After incubation, the protease solution was removed and the explants were transferred to a flask containing a 0.2% collagenase solution (Merck) in serum-free D-MEM, and kept under oscillation (120 rpm) for 2 h at 37 °C.

After the second digestion, the solution was filtered by gravity, sequentially using 100 μm and 40 μm filters to eliminate any residual tissue pieces.

Complete D-MEM medium was added to the cell suspension to inactivate collagenase; the cell suspension was centrifuged at 400 xg for 7 min to allow cells to form a tight pellet. After removing the supernatant, the cell pellet was resuspended in complete D-MEM medium and then chondrocytes were counted using a Bürker hemocytometer and cell viability (> 95%) was assessed by Trypan Blue (0.1%) exclusion assay. The obtained cells were seeded in cell culture dishes, where they grow as adherent cells, or encapsulated in sodium alginate beads.

### 2.4 Cell cultures in 2D

Cells isolated from the tissue were seeded in 12-well cell cultures plates for P1 cultures and in cell culture dishes (57 cm^2^) for amplification for the subsequent passages (P2 and P3 cultures).

After confluence was reached in P1 cell cultures, cells were trypsinized, counted using the Bürker hemocytometer and re-seeded in plates at the density of 2 × 10^4^ cells/cm^2^ to obtain chondrocytes at two serial culture passages (P2 and P3).

Also chondrocytes at P2 and P3 were then seeded in 12-well cell cultures plates at the density of 2 × 10^4^ cells/cm^2^ in complete D-MEM medium and incubated at 37 °C, 5% CO_2_ (1, 3, 24).

### 2.5 Cell cultures in alginate beads

To perform 3D cultures, cells were seeded into alginate beads. Briefly, isolated chondrocytes were resuspended in a sodium alginate solution (1.2% in 0.9% NaCl) at 4 × 10^6^ cells/mL. The solution was dripped using a 22-gauge needle syringe into a CaCl_2_ solution (102 mM, Merck) kept in gentle agitation. The droplets of the alginate cell suspension polymerize very quickly when in contact with the CaCl_2_ solution, trapping the chondrocytes inside. After bead polymerization, the CaCl_2_ solution was removed, the beads were washed 3 times with sterile 0.9% NaCl and then transferred into 12-well cell cultures plates in complete D-MEM medium and incubated at 37 °C, 5% CO_2_.

### 2.6 Experimental conditions

Both cell cultures in 2D and chondrocytes embedded in alginate beads were incubated for 2 weeks at 37 °C, 5% CO_2_, with 20% O_2_ (normoxia) or 5% O_2_ (hypoxia) in a Water Jacketed Incubator series 3 (ThermoFisher Scientific, Waltham, MA, USA) and in complete D-MEM medium at three different osmolarity values (280, 380, and 480 mOsm/L). Three independent experiments were performed.

### 2.7 Morphology

At the defined experimental time-points, cells in 2D and alginate beads were fixed with 4% paraformaldehyde (PFA, Merck) in PBS for 40 min at room temperature. After fixation, the samples were washed to eliminate any residues of PFA. Alginate beads were pre-embedded in 100 μL of a 2% agarose solution (Merck) in distilled water to preserve the 3D culture and facilitate handling; the samples were then dehydrated in increasing alcohol concentrations, clarified in xylene, and paraffin-embedded; 5-μm-thick histological sections were obtained using a rotary microtome (Slee Cut 6,062, Slee Medical, Mainz, Germany). The sections of alginate beads and the monolayer cell cultures were stained with Masson’s trichrome (with Aniline-Blue; Bio-Optica, Milano, Italy). Microphotographs were acquired by using a Nikon Digital Sight System (Nikon, Japan).

### 2.8 MTT colorimetric assay

At the defined experimental time-points, an MTT colorimetric assay (Merck) was performed. The MTT assay was performed by adding 20 μL of a 5 mg/mL MTT [3-(4,5-dimethylthiazol-2-yl)-2,5-diphenyltetrazolium bromide] solution to cells cultured in 100 μL of medium, followed by incubation for 4 h at 37 °C. Afterwards, 100 μL of a solubilization solution (10% SDS in 0.01 M HCl; Merck) were added and incubated O/N at 37 °C. The absorbance of the solution was read at 540 nm by using a VICTOR® Nivo™ Multimode Microplate Reader (Perkin Elmer). The absorbance read in wells with medium and MTT without cells was used as control.

### 2.9 Quantification of glycosaminoglycans (GAGs)

At the defined experimental time-points, the culture medium was collected and kept a − 80 °C until analysis. A volume of 20 μL of medium or 20 μL of standard (C-4-S chondroitin sulphate solution [Merck] ranging from 0 to 5 μg) was added in duplicate to a 96-well plate. A volume of 200 μL of a 1,9-dimethylmethylene blue (DMMB; Merck) solution at pH 1.5 (16 mg DMMB in 1 litre of water containing 0.03 g glycine, 1.6 g NaCl and 95 mL of 0.01 M acetic acid) was added to wells and absorbance was immediately read at 525 nm by using a VICTOR® Nivo™ Multimode Microplate Reader (Perkin Elmer) (25–27); the GAG amount value was normalized to the DNA content.

### 2.10 Gene expression analysis

To evaluate gene expression of specific markers related to the chondrocyte phenotype based on the tested conditions, real-time quantitative PCR (qPCR) analysis was performed. RNA extraction was performed using the TRI-reagent® Kit (ThermoFisher Scientific), according to the manufacturer’s instructions. The first step in using TRI-Reagent® differs based on the culture system used:

(a) for extraction from adherent cells, the medium was removed from wells and 1 mL of TRI-reagent® was added per well. Scraping was performed to recover cells;
(b) for extraction from beads, the medium was removed and the beads were dissolved in a volume of citrate buffer (55 mM Na-citrate, 50 mM EDTA, 0.15 M NaCl, pH 7.4) equal to three times the volume occupied by the beads. After 15 min, the solution was centrifuged at 400 xg for 8 min, the supernatant was discarded, and the pellet was washed with PBS and lysed with 1 mL of Tri-reagent®.

The homogenates obtained were processed according to the manufacturer’s instructions. RNA samples were resuspended in DEPC water. To valuate purity and concentration, 1 μL of RNA was quantified with a NanoDrop spectrophotometer (ThermoFisher Scientific), using DEPC water as blank. RNA integrity and quality were assessed by using an Agilent Bioanalyzer 2,100 and RNA 6000 Labchip kit (Agilent Technologies, USA).

For reverse transcription (RT), the High Capacity cDNA Reverse Transcription kit (ThermoFisher, Scientific) was used a*ccording* to the *manufacturer’s* instructions. One microgram of total RNA was resuspended in DEPC water to a final volume of 10 μL and mixed with 10 μL of RT master mix.

For real-time qPCR, the Power-Up SYBR Green Master Mix kit (ThermoFisher Scientific) was used along with a set of primers specific for each gene (Table 1) and the cDNA samples were added in a final reaction volume of 20 μL. According to the thermal profile, samples were kept at 95 °C for 20 s and then subjected to 40 cycles consisting of denaturation at 95 °C for 3 s, followed by annealing/extension at 60 °C for 30 s. The reaction was performed by using a StepOne thermocycler (StepOne software v. 2.3 Applied Biosystems; ThermoFisher Scientific). SYBR Green fluorescence incorporation was acquired at the end of the extension step. In each experiment, to check specificity of amplification, a no-RT control and a no-template control (NTC) were included. The 18S gene was used as an endogenous control selected among other tested reference genes (i.e., GAPDH, *β*-2MG, and HPRT) due to minimal intra−/inter-assay variation. The data obtained from qPCR were analyzed according to the 2^-∆∆Ct^ method (28); in the present study, the expression level of each gene was normalized to the amount of 18S and expressed as relative quantities (RQ) calculated with reference to the cells grown in control medium incubated in normoxia at the P1 passage. Data were presented as mean ± standard deviation (SD) of three independent experiments, each performed in duplicate.

**Table 1:** Primer pair sequences used for qPCR amplification of chondrocyte markers in 2D cell cultures and 3D alginate beads subjected to variations in osmolarity and oxygen tension.

Target gene	GenBank accession number	Primer sequence FOR-REV (5′-3′)	Concentration (nM)	References
*COLL1A1*	AF_034691	AGA AGA AGA CAT CCC AGC AGT CA CAG GGC TCG GGT TTC CAT A	500 500	Ravanetti et al. (32)
*COLL2A1*	NM_001081764	GGA TGG CTG CAC GAA ACA C CAG GCG CGA GGT CTT CTG	300 300	Ravanetti et al. (32)
*ACAN*	XM_001917528	GAC CAC TTT ACT CTT GGC GTT TG GTC AGG GTC TGA AAC GTC TAC TGA	500 500	Ravanetti et al. (32)
*SOX9*	XM_001498424	CAG GTG CTC AAG GGC TAC GA GAC GTG AGG CTT GTT CTT GCT	300 300	Ravanetti et al. (32)
*RUNX2*	XM_001502519	CTG TGG TTA CTG TCA TGG CG TCG TTG AAC CTT GCC ACT TG	300 300	Ravanetti et al. (32)
*HIF-1α*	XM_005605527.1	AAG CTT TGG ATG GTT TTG TTA GTA CTT CCA TGT TGC AGA CTT TAT	300 300	Ravanetti et al. (32)
*BGT1* (*SLC6A12*)	XP_070126948.1	ATC CTC GCC ATC TCT GTC AC GTG CCA CTG CAA GCG TAA TA	250 250	De Angelis et al. (24)
*18S*	AJ_311673	ATG CGG CGG CGT TAT TCC GCT ATC AAT CTG TCA ATC CTG TCC	300 300	Kayis et al. (56)

### 2.11 Statistical analysis

**Figure 1:**
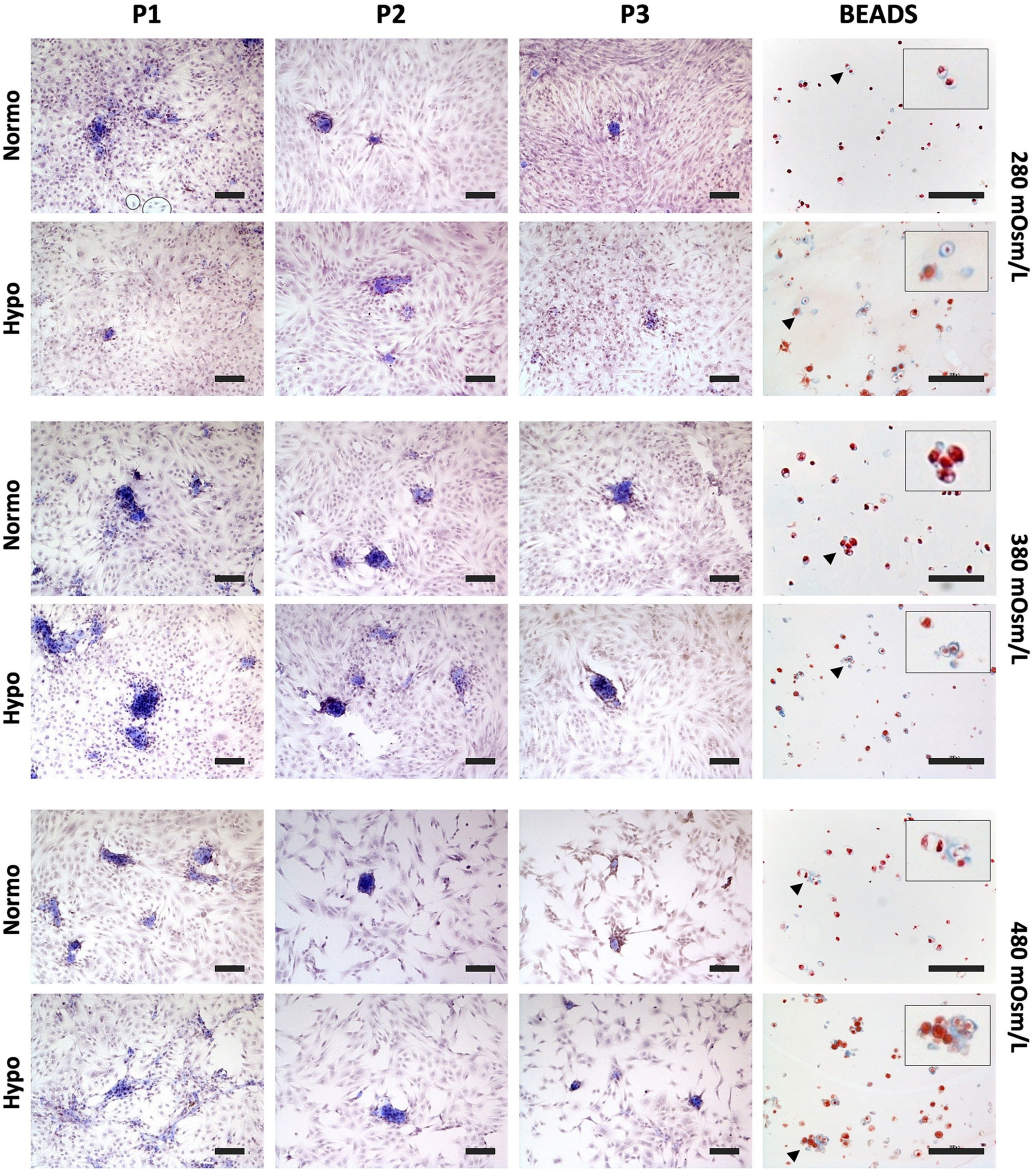
Representative light micrographs of chondrocytes cultured on plastic (P1–P3) or embedded in alginate beads under normoxic (Normo) or hypoxic (Hypo) conditions at medium osmolarities of 280, 380, or 480 mOsm/L for 2 weeks. Samples were stained with Masson’s trichrome. Original magnification, ×20. High-magnification insets highlight positively stained cell clusters (arrows). Scale bar = 100 μm.

Experimental data are presented as mean ± standard deviation (SD) of three independent biological experiments. Statistical analyses were conducted separately within each defined experimental condition (e.g., 2D and 3D culture systems) to evaluate the effect of treatments within a fixed biological context. Given that 2D and 3D culture systems represent biologically distinct experimental contexts, statistical analyses were performed separately within each system to evaluate the effects of oxygen tension and osmolarity at a defined passage. Accordingly, differences among treatment groups within each fixed biological level were analyzed using one-way analysis of variance (ANOVA), followed by Dunnett’s multiple comparison test versus the respective control group (GraphPad Prism v.7.0, La Jolla, CA, USA). A *p*-value < 0.05 was considered statistically significant. Statistical significance among treatments is reported within figures (1).

## 3 Results

### 3.1 Morphology

Morphological analysis (Figure 1) revealed that, under hyperosmolar conditions, both in normoxia and hypoxia, cell density was markedly reduced, with a more pronounced decrease observed in P3 cells.

At P1 and P2, exposure to hyperosmolar media (380 and 480 mOsm/L), particularly under hypoxic conditions, induced evident morphological changes compared to the 280 mOsm/L condition (Figure 1). Alongside flattened and elongated cells with a fibroblast-like morphology, a population of roundish cells organized into clusters was observed. In contrast, these cell clusters were rarely detected in normoxic cultures and in P3 cells (Figure 1). Masson’s trichrome staining revealed extracellular matrix production predominantly localized within the clustered cell areas and in the pericellular space of cell clusters.

In the 3D alginate bead culture system, chondrocytes displayed a predominantly round morphology and were homogeneously distributed. However, under hyperosmolar conditions (380 and 480 mOsm/L), particularly in hypoxia, clusters composed of two or more cells were frequently observed. Consistently, Masson’s trichrome staining demonstrated matrix accumulation in the pericellular space surrounding these cell clusters (Figure 1).

### 3.2 MTT assay

**Figure 2:**
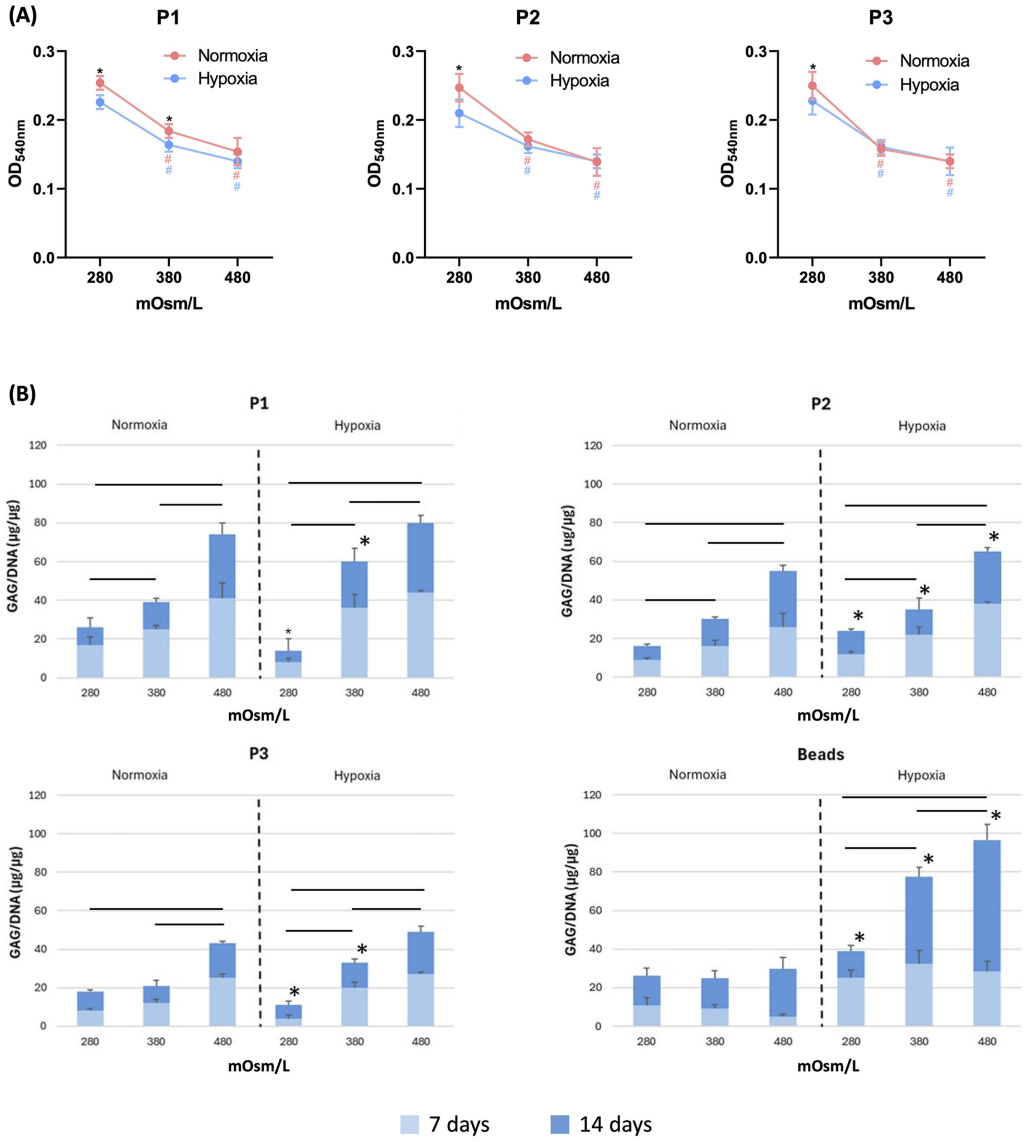
**(A)** MTT assay of articular chondrocyte cultures maintained for 14 days in adhesion in cell cultures dishes at passages P1, P2, and P3. Cells were cultured in media with different osmolarities (280, 380, and 480 mOsm/L) under normoxic or hypoxic conditions. The hash symbols (#) indicate statistically significant differences between osmolarity conditions within the same cell passage. Asterisks (*) indicate statistically significant differences between normoxic and hypoxic conditions at the same passage and osmolarity (*p* < 0.05). **(B)** Quantification of glycosaminoglycans (GAGs) released in the supernatant after 7 and 14 days of chondrocyte culture. Articular chondrocytes were cultured either in adherent conditions (P1, P2, and P3) or in 3D alginate beads, under normoxia or hypoxia and at different medium osmolarities (280, 380, and 480 mOsm/L). Horizontal bold bars indicate statistically significant differences among osmolarity conditions within the same passage. Asterisks (*) indicate statistically significant differences between normoxic and hypoxic conditions at the same passage and osmolarity (*p* < 0.05).

Cell viability/proliferation, assessed by MTT assay (Figure 2A), progressively decreased with increasing medium osmolarity in all cell passages (P1–P3) and under both normoxic and hypoxic conditions. In normoxia, MTT values were significantly reduced at 380 and further at 480 mOsm/L compared to 280 mOsm/L across all passages. Hypoxic culture conditions significantly reduced MTT values at 280 mOsm/L compared to normoxia in P1, P2, and P3. Under hypoxia, exposure to hyperosmolar media (380 and 480 mOsm/L) resulted in a further decrease. This reduction was more pronounced at P1 and P2, whereas P3 cells showed comparable low MTT values at 380 and 480 mOsm/L.

### 3.3 GAG production

GAG production in the cell culture supernatant was increased by incubation in hyperosmolar media across all cell passages (Figure 2B). Particularly in P1, hypoxic conditions significantly enhanced GAG production in cells cultured at 380 mOsm/L compared to controls at 280 mOsm/L. In P2 the hypoxic condition significantly increased the GAG production at all the osmolarity concentration. In P3, the hypoxic effect on GAG production compared to normoxia condition was evident only at 280 and 380 mOsm\L. In the supernatant of cells cultured in alginate beads, GAG levels were higher in hypoxia compared to that quantified in the normoxia conditions, at all the osmolarity concentrations. Furthermore, in the alginate bead system, the effect of the hyperosmolar on GAG production was evident only under hypoxic conditions (Figure 2B).

### 3.4 Gene expression of differentiation markers in chondrocytes cultured in 2D and in alginate beads

**Figure 3:**
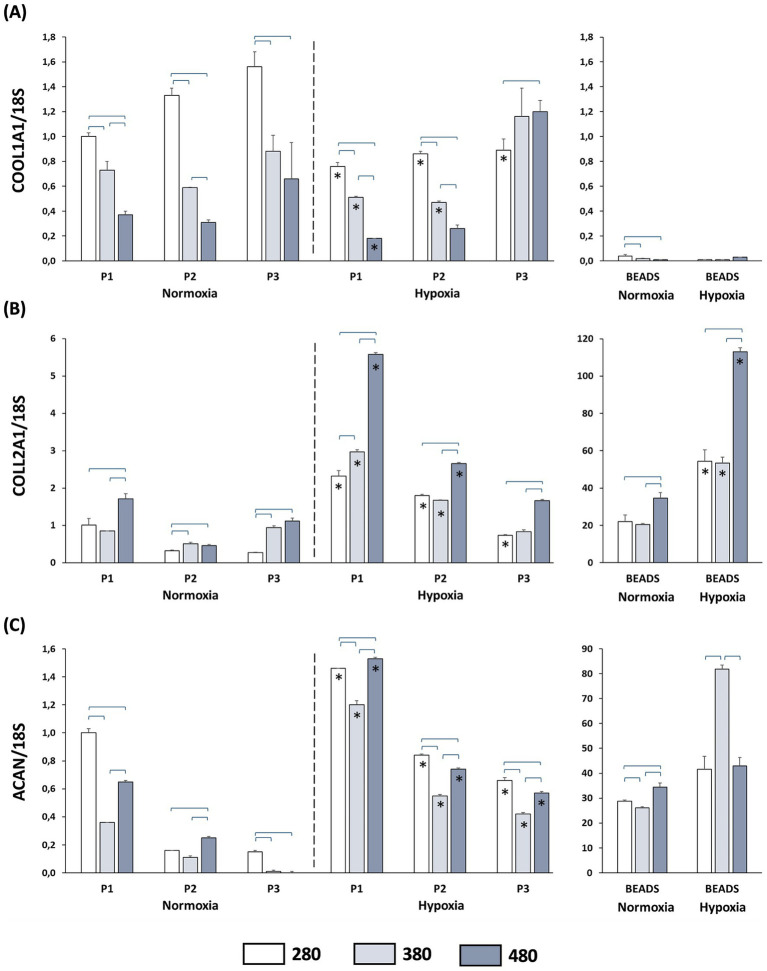
Gene expression of extracellular matrix–related genes in articular chondrocytes cultured in media with different osmolarities (280, 380, and 480 mOsm/L) and under normoxic or hypoxic conditions. Articular chondrocytes were cultured for 14 days either under adherent conditions on tissue culture dishes at passages P1, P2, and P3 or in 3D alginate bead culture. **(A)** Relative expression of *COLL1A1*, **(B)** relative expression of *COLL2A1*, and **(C)** relative expression of *ACAN*. Horizontal brackets indicate statistically significant differences among osmolarity conditions within the same passage, while asterisks indicate statistically significant differences between normoxic and hypoxic conditions at the same passage and osmolarity (*p* < 0.05).

As expected in dedifferentiation process, *COLL1A1* gene expression increased with cell culture passages at 280 mOsm/L (from P1 to P3) in normoxia. When cells were cultured in hyperosmolar media at 380 and 480 mOsm/L *COLL1A1* gene expression was reduced (Figure 3A) compared to 280 mOsm in all passages. Under hypoxia, *COLL1A1* expression in P1 and P2 was further decreased at all osmolarities, while at P3 the reduction was observed only at 280 mOsm/L. In alginate beads cultures, *COLL1A1* expression was consistently low under both normoxic and hypoxic conditions (Figure 3A).

*COLL2A1* and *ACAN* gene expression analysis showed, as expected, a reduction when chondrocytes were cultured at passages P2 and P3 compared to P1 (Figures 3B,C). Under normoxic conditions, the incubation in the hyperosmolar medium (particularly at 480 mOsm/L) resulted in increased *COLL2A1* expression (Figure 3B) at all passages compared to control medium at the corresponding passage. Culture under hypoxic condition further enhanced *COLL2A1* expression at all passages, and the association with hyperosmolar media induced higher expression levels compared to control conditions. Analysis of *ACAN* gene expression (Figure 3C) showed an increase under normoxia conditions when P2 chondrocytes were incubated in medium at 480 mOsm/L. Hypoxic incubation induced a significant increase in *ACAN* expression at all passages and at all osmolarities compared to normoxia conditions. When chondrocytes were cultured in alginate beads under normoxia, incubation in medium at 480 mOsm/L increased *COLL2A1* and *ACAN* expression. Hypoxic conditions further enhanced the expression of both genes compared to normoxia at all osmolarities. With the greatest increase observed at 480 mOsm/L (Figures 3B,C).

**Figure 4:**
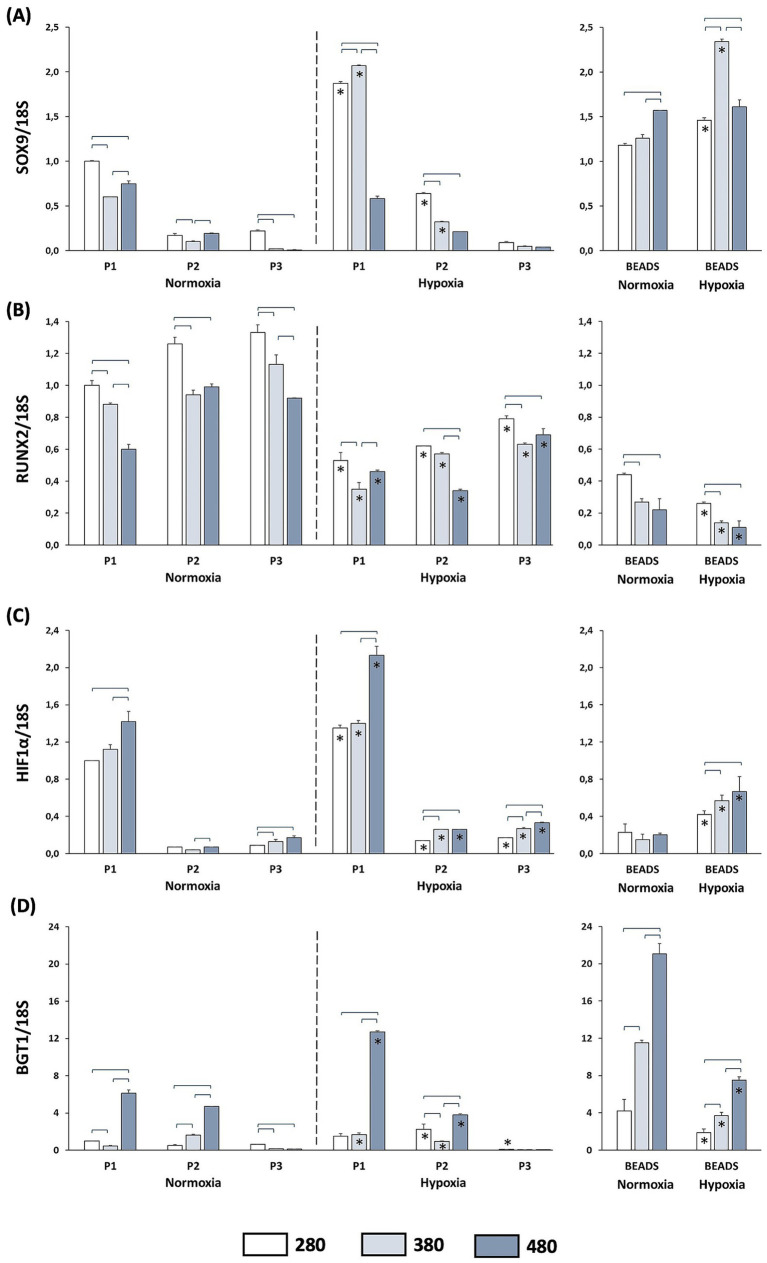
Gene expression of extracellular matrix–related genes in articular chondrocytes cultured in media with different osmolarities (280, 380, and 480 mOsm/L) and under normoxic or hypoxic conditions. Articular chondrocytes were cultured for 14 days either under adherent conditions on tissue culture dishes at passages P1, P2, and P3 or in 3D alginate bead culture. The relative expression of *SOX9*, *RUNX2*, *HIF1α*, and *BGT1* is shown in panels **(A–D)**, respectively. Horizontal brackets indicate statistically significant differences among osmolarity conditions within the same passage, while asterisks indicate statistically significant differences between normoxic and hypoxic conditions at the same passage and osmolarity (*p* < 0.05).

*SOX9* gene expression was reduced when chondrocytes were cultured at passages P2 and P3 compared to P1 (Figure 4A). Under normoxic conditions, incubation in hyperosmolar media lead to a decrease in *SOX9* expression especially at P1 and P3, compared to control medium at the corresponding passage. Hypoxic incubation increased *SOX9* expression at P1 and P2, but not at P3, compared to normoxic conditions. However, exposure to hyperosmolar media at 380 mOsm/L (P2) and 480 mOsm/L (P1 and P2) attenuated *SOX9* expression under hypoxia. When chondrocytes were cultured in alginate beads under normoxia, incubation in medium at 480 mOsm/L increased *SOX9* expression. Hypoxic culture in combination with hyperosmolar media in alginate beads increased *SOX9* expression (Figure 4A).

Chondrocytes culture at passages P2 and P3 increased *RUNX2* gene expression compared to P1 (Figure 4B). Incubation in hyperosmolar medium (380 and 480 mOsm/L) resulted in a reduction of *RUNX2* expression at all passages. Under hypoxic conditions, *RUNX2* expression was decreased at all three passages compared to normoxia, with a more pronounced effect observed in hyperosmolar media. In chondrocytes cultured in alginate beads, hyperosmolar conditions induced a slightly reduction in *RUNX2* gene expression, whereas hypoxia led to a significant decrease across all osmolarity conditions when compared with normoxia (Figure 4B).

*HIF-1α* gene expression was markedly reduced at passages P2 and P3 compared to P1 (Figure 4C). Under normoxic conditions, P1 chondrocytes cultured in hyperosmolar medium (480 mOsm/L) exhibited higher *HIF-1α* expression levels than those maintained at lower osmolarities, an effect that was further enhanced under hypoxic conditions. In addition, incubation of P2 and P3 chondrocytes in hyperosmolar media under hypoxia resulted in an upregulation of *HIF-1α* expression. Experiment performed in alginate beads demonstrated that hypoxia significantly increased *HIF-1α* expression compared to normoxia; in this setting, significant differences were observed between the 280 mOsm/L condition and the hyperosmolar media (Figure 4C).

A significant upregulation of gene expression of the transporter for betaine (*BGT1*) was observed at 480 mOsm/L at P1 and P2, but not at P3 (Figure 4D). In P1 chondrocytes, the upregulation observed at 480 mOsm/L was increased when cells were incubated in the hypoxia condition; furthermore, in hypoxia, an increase of gene expression of the betaine transporter was observed at P1 380 mOsm/L compared to cells incubated in normoxia. In chondrocytes grown in the 3D culture system, *BGT1* gene expression showed a progressive significant upregulation at 380 and 480 mOsm/L compared to 280 mOsm/L. Exposure to hypoxic conditions attenuated this upregulation across all osmolarities compared to normoxia although the progressive increase from 280 mOsm/L to 480 mOsm/L was preserved (Figure 4D).

**Figure 5:**
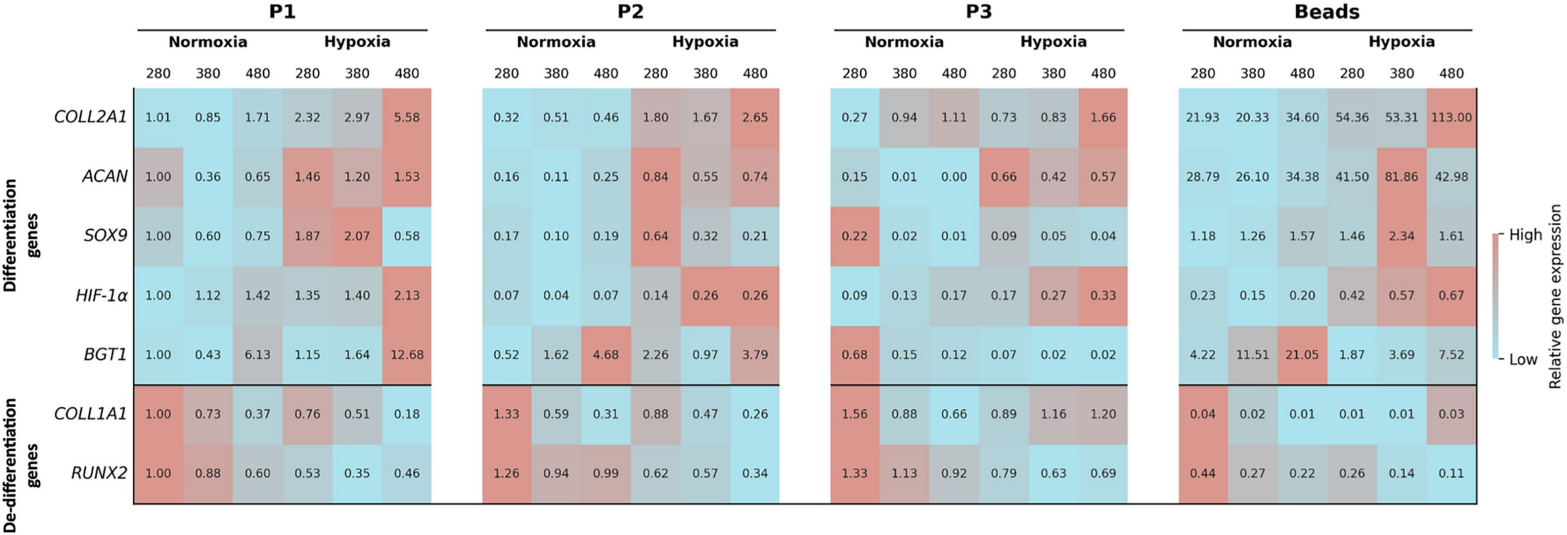
Heatmap-based gene expression analysis (RT-PCR) of extracellular matrix-related, transcription factor, and transporter genes in articular chondrocytes. Cells were cultured for 14 days in media with different osmolarities (280, 380, and 480 mOsm/L) under normoxic or hypoxic conditions. Chondrocytes were maintained either under adherent conditions (passages P1, P2, and P3) or in 3D alginate bead culture. Genes are ordered to highlight expression patterns relative to the experimental conditions. High expression levels are highlighted in red, while low expression levels are shown in light blue.

Heatmap-based gene expression analysis, derived by RT-PCR results, revealed distinct patterns associated with differentiation and de-differentiation phenotypes (Figure 5). Under normoxic conditions, differentiation-associated genes displayed overall low expression levels across all monolayer passages (P1–P3) as well as in alginate bead cultures, whereas de-differentiation markers exhibited relatively higher expression, particularly at the lowest osmolarity (280 mOsm/L). In contrast, hypoxic conditions were characterized by a marked upregulation of differentiation genes, especially at higher osmolarities (380 and 480 mOsm/L), concomitant with reduced expression of de-differentiation markers. Notably, although differentiation markers were consistently expressed at lower levels under normoxia compared to hypoxia across all passages, alginate bead cultures showed an overall higher expression of differentiation genes compared to monolayer cultures, irrespective of O_2_ conditions (Figure 5).

## 4 Discussion

*In vitro* de-differentiation is a major limitation for cell-based therapies in cartilage repair because during this process, adult chondrocytes progressively acquire a fibroblast shape and lose their capability to produce ECM (6). Therefore, it is crucial to define the most suitable culture conditions that can minimize chondrocyte *in vitro* de-differentiation. In this view, our results demonstrate that modulation of cartilage-like microenvironmental conditions attenuated the loss of the chondrocyte phenotype, in particular, during the early phases of de-differentiation, showing a synergistic action of hypoxia and hyperosmolarity. When de-differentiation was more advanced, this effect became less pronounced and the cellular responsiveness to hypoxia and hyperosmolarity progressively diminished, despite significant effects on *COLL2A1* and *RUNX2* expression were still observed.

Numerous studies have investigated the effects of a lower oxygen tension (1–5% O_2_ vs. 20% O_2_) on the chondrocyte phenotype and ECM (29–31). More recently, we have demonstrated that hypoxia is a micro-environmental condition that reduces chondrocyte de-differentiation during *in vitro* expansion (32).

The impact of osmolarity changes on chondrocyte differentiation and metabolism has received more attention in the recent years but remains less understood, especially in terms of mechanisms of the biological response and phenotype differentiation/de-differentiation (33–39).

To date, very few studies have addressed the combined effects of these two microenvironmental factors—O_2_ tension and osmolarity—on chondrocyte differentiation, both during the expansion phase in monolayer (2D) culture and in 3D culture systems such as alginate beads. For instance, Jurgens and co-workers (40) examined the effects of hyperosmotic and/or hypoxic conditions on adipose stem cells (ASCs) and demonstrated that in a 3D micro-environment, this combination was able to induce chondrogenesis to the same extent as TGF-β1. More recently, Sieber and co-workers (41) showed that the combination of cartilage-specific O_2_ tension and osmolarity have a positive effect on the porcine chondrocyte phenotype in a scaffold-free 3D culture system. In our study we used equine articular cartilage because horses naturally develop degenerative joint disease under physiological loading conditions (e.g., OA) and their cartilage thickness, zonal architecture, and collagen fibril organization closely resemble those of humans. Therefore, equine cartilage is often considered particularly suitable for translational studies (42–44).

To our knowledge, this is the first study to investigate the behavior of adult horse articular chondrocytes under combined hypoxic and hyperosmotic conditions during progressive in vitro de-differentiation (across the serial passages P1, P2, and P3), in comparison with a 3D culture system. In the present study, articular chondrocytes were cultured simultaneously under standard culture conditions (20% O_2_—hyperoxia for chondrocytes—and 280 mOsm/L) and under physicochemical conditions mimicking the native cartilage environment (5% O_2_—physoxia for chondrocytes—and osmolarity ranging from 380 to 480 mOsm/L). This approach demonstrated that modulating these microenvironmental parameters positively influences the expression of phenotypic markers, particularly during the early phase of de-differentiation, and to a lesser extent during later passages, when de-differentiation becomes more established.

In a previous study, we characterized a model of chondrocyte de-differentiation in horses by analyzing gene expression of key chondrogenic markers during serial monolayer passages and as a function of cumulative population doubling levels (PDLs) (1). As expected, the expression of all chondrocyte phenotypic markers was already markedly reduced in primary monolayer culture (P1) compared to freshly isolated chondrocytes and to chondrocytes cultured in a 3D model (alginate beads). Specifically, in a cell model of proliferating primary chondrocytes, we separately demonstrated that hypoxia (32) and osmolarity (24) can specifically and significantly reduce the chondrocyte phenotype loss. As demonstrated in the previous work (1) during chondrocyte de-differentiation in a monolayer culture under normoxic conditions at 280 mOsm/L, *COLL1A1* expression progressively increased from P1 to P3. In contrast, *COLL1A1* expression was markedly reduced in the 3D alginate bead culture, consistent with the 3D microenvironment ability to support a differentiated chondrocyte phenotype (3, 24). Under hyperosmolar conditions (380 and 480 mOsm/L), *COLL1A1* expression decreased, suggesting that physiological hyperosmolarity can reduce chondrocyte de-differentiation (39). Furthermore, under hypoxic conditions at 280 mOsm/L, *COLL1A1* expression was reduced, and notably, the combination of hypoxia and hyperosmolarity led to an even greater suppression of *COLL1A1* expression across culture passages. Accordingly, *RUNX2*, marker of de-differentiation, was more highly expressed under normoxic conditions, while its expression decreased in hypoxia and in response to increasing osmolarity, which is supportive of differentiation. As expected, the lowest expression levels were observed in the 3D cultures, particularly under hypoxic conditions (12, 45). As expected, in the monolayer culture, a significant and sustained down-regulation of *COLL2A1* was observed during the early stages of de-differentiation, and this was maintained throughout the subsequent culture passages (1). Both hypoxia and hyperosmolarity, when applied independently, promoted higher expression of *COLL2A1* compared to normoxic and iso-osmotic conditions (24, 32). A synergistic effect of hypoxia and hyperosmolarity became evident when *COLL2A1* gene expression was analyzed. In hypoxia and hyperosmolar (380 and 480 mOsm/L) condition, significantly elevated and sustained *COLL2A1* expression was detected during the early stages of de-differentiation, and this enhancement was maintained throughout the subsequent passages. Furthermore, in the differentiated 3D culture system (beads), a synergistic effect of hypoxia and hyperosmolarity on *COLL2A1* gene expression was also evident. Specifically, under normoxic conditions, *COLL2A1* expression was strongly upregulated at P1 at 280 and 380 mOsm/L and further elevated at 480 mOsm/L. Under hypoxic conditions, *COLL2A1* expression was even more markedly induced at 280 and 380 mOsm/L, and reached a peak at 480 mOsm/L with an approximately 110-fold increase, indicating a microenvironment strongly inductive to chondrogenic differentiation.

In contrast, the expression of *ACAN* and *SOX9* did not appear to be significantly affected by the combined influence of hypoxia and osmolarity during *in vitro* de-differentiation. Particularly at P3, the marked reduction in ACAN gene expression highlights that dedifferentiation may negatively affect the cellular response to increased extracellular osmolarity. Consistently, the 3D culture system under low-oxygen conditions further enhanced *ACAN* gene expression across all osmolarity levels. In the bead-based 3D culture, a synergistic effect on *ACAN* and *SOX9* expression was particularly evident at an osmolarity of 380 mOsm/L, in agreement with previous findings (41). Regarding GAG release into the culture medium, an overall increase was observed across all passages under hyperosmolar conditions. A combined effect with hypoxia was detected only at passage P1, but this interaction was not maintained as de-differentiation progressed. Notably, in differentiated chondrocytes cultured in the 3D system, a clear synergistic effect of hyperosmolarity and hypoxia was evident. Morphological analyses qualitatively supported these findings, showing an apparent increase in extracellular matrix deposition around cell clusters, as evidenced by Masson’s trichrome staining. Although this staining does not allow specific identification of individual matrix components, the overall pattern was indicative of increased matrix deposition. These observations are in line with previous reports (41, 46, 47). Proliferation and viability data indicated that hyperosmolarity and hypoxia are associated with a decrease in MTT assay readouts, reflecting a reduction in the proliferative potential of chondrocytes that depends on culture passage and osmolarity. Hypoxia further promotes differentiation in the early passages (P1 and P2), resulting in the formation of characteristic cell clusters. This cellular behavior is consistent with, and further clarifies, the potential synergistic effects of hyperosmotic and hypoxic microenvironments (24, 32).

Hypoxia-inducible factor-1 alpha (*HIF-1α*) plays a crucial role in chondrocyte survival within their naturally hypoxic environment by promoting a metabolic shift toward glycolysis. This shift enables chondrocytes to generate energy despite limited oxygen availability and reduces the overall oxygen consumption, further supporting cell survival (48–50). The decrease of *HIF-1α* gene expression during the de-differentiation phase is limited in conditions of hypoxia especially combined with hyperosmolarity and this synergistic effect was also well evident in the condition of differentiated chondrocytes (3D culture).

In a previous work (24) we examined gene expression of the betaine-*γ*-aminobutyric acid transporter 1 (*BGT1*) in horse cultured articular chondrocytes exposed to increased osmolarity (≈ 380–480 mOsm/L) resulting in a down-regulation of de-differentiation markers (e.g., *COLL1A1* and *RUNX2*) and up-regulation of differentiated chondrocyte markers such as *COLL2A1* and *ACAN*. In this context, *BGT1* levels remained persistently elevated in both proliferating and differentiated chondrocytes under hyperosmolar conditions, suggesting that BGT1, as osmolyte transporter, may play a supportive role in promoting or stabilizing the differentiated articular chondrocyte phenotype through volume regulatory mechanisms in a hyperosmotic environment which is relevant to cartilage structure and physiology. In the present study we confirm that *BGT1* gene expression typically increased in response to hyperosmolarity in all culture conditions (24) and was lost only in advanced stage of de-differentiation.

To our knowledge, very few previous studies have investigated the combined effect of hypoxia and osmolarity on chondrocyte biology. Notably, this is the first study to examine de-differentiation processes under concomitant hypoxic and hyperosmotic conditions using adult articular chondrocytes, both during *in vitro* de-differentiation in monolayer culture and in a re-differentiated 3D context (40, 41). First of all, under conditions promoting de-differentiation, our data show that the combined application of hypoxia and increased osmolarity more effectively mimics the native cartilage microenvironment, supporting phenotypic stability and maintaining ECM integrity. At the molecular level, this environment seems to support the maintenance of key chondrogenic markers, including *SOX9*, *COLL2A1*, and *ACAN*, while limiting the expression of fibroblastic markers associated with de-differentiation, *COLL1A1* and *RUNX2*. In addition, recent advances in 3D culture systems confirm that embedding chondrocytes in physiologically relevant matrices enhances cartilage-specific gene expression and matrix deposition compared to 2D expansion alone; priming strategies during expansion have also shown promising results in preserving phenotype and enhancing matrix production upon subsequent 3D culture (51, 52). All these findings underscore the central role of the culture microenvironment in regulating chondrocyte plasticity and functional outcomes for engineered tissue constructs. These insights are directly relevant to translational cartilage repair strategies, including autologous chondrocyte implantation (ACI) and matrix-assisted autologous chondrocyte implantation (MACI). Such procedures require extensive cell expansion prior to implantation, during which loss of chondrogenic phenotype remains a major challenge. The differentiation status of chondrocytes at the time of implantation is a critical determinant of the quality and durability of the repair tissue: cells that maintain or reacquire a differentiated phenotype more likely generate hyaline-like cartilage rich in type 2 collagen and proteoglycans, whereas extensively de-differentiated cells tend to produce fibro-cartilaginous tissue with inferior biomechanical properties (53, 54). Thus, strategies that modulate the microenvironment—such as controlled oxygen tension, physiological osmolarity, and optimized 3D culture platforms—may not only mitigate de-differentiation but also enhance the regenerative capacity of cellular constructs. Preserving a functional chondrocyte phenotype prior to transplantation could improve graft integration and long-term outcomes in clinical cartilage repair (40, 41).

As a conclusion, the results obtained in this study may have a significant impact on tissue engineering applications using articular chondrocytes. Although numerous pre-clinical and clinical studies have confirmed the importance of optimizing chondrocyte differentiation using both adult differentiated chondrocytes and MSCs, most existing clinical studies provide low levels of evidence. Therefore, as future prospective, randomized controlled trials are needed to confirm their ability to promote cartilage repair.

## Data Availability

The original contributions presented in the study are included in the article/supplementary material, further inquiries can be directed to the corresponding author.
